# Dr. Salmon’s Report on Government Meat-inspection

**Published:** 1898-06

**Authors:** 


Dr. D. E. SALMON'S presentation, in the Department of Agriculture's year-book, of the work done by the veterinary division of the Bureau of Animal Industry is filled with interesting data marking the progress and development of this department and demonstrating its importance to the welfare of those engaged in the pursuits of animal industry. He well says that this is one division of national public work that has saved and returned thousands of dollars to our people for hundreds expended. Every veterinarian in the land, especially in Illinois, where the great central slaughtering-houses are located, with their meat-inspection service, should read this excellent presentation of the subject carefully, and he will find himself better equipped to advance the best reasons why this inspection service should be carried out along all the lines of food-products gathered from our animal husbandry. Dr. Salmon well sustains the confidence of the profession as its chief in this work of our national government, and should continue to receive their warm support in the broadening of this all-important work.

